# Independent and inter-dependent immunoregulatory effects of NCF1 and NOS2 in experimental autoimmune encephalomyelitis

**DOI:** 10.1186/s12974-020-01789-2

**Published:** 2020-04-11

**Authors:** Jianghong Zhong, Anthony C. Y. Yau, Rikard Holmdahl

**Affiliations:** 1 Medical Inflammation Research, Department of Medical Biochemistry and Biophysics, Karolinska Institute, 17177 Stockholm, Sweden; 2 Beijing Advanced Innovation Center for Big Data-Based Precision Medicine, Beihang University, Beijing, 100083 China

**Keywords:** Experimental autoimmune encephalomyelitis, NCF1, NOS2, Interleukin-1β, Neutrophil

## Abstract

**Background:**

Increasing evidence has suggested that a single nucleotide polymorphism in the *Ncf1* gene is associated with experimental autoimmune encephalomyelitis (EAE). However, the mechanisms of NCF1-induced immunoregulatory effects remain poorly understood. In this study, we focus on NCF1 deficiency-mediated effects on EAE in NOS2 dependent and independent ways.

**Methods:**

To determine the effects of NCF1 and NOS2 during EAE development, we have established recombinant mouse strains deficient at NCF1 and/or NOS2 in a crossbreeding system. Different strains allow us to examine the entire course of the disease in the *Nos2*-null mice bearing a *Ncf1* gene that encodes a mutated NCF1, deficient in triggering oxidative burst, after immunization with recombinant myelin oligodendrocyte glycoprotein (MOG)_79-96_ peptides. The peptide-induced innate and adaptive immune responses were analyzed by flow cytometry.

**Results:**

NCF1-deficient mice developed a reduced susceptibility to EAE, whereas NCF1-NOS2 double-deficient mice developed an enhanced EAE, as compared with NOS2-deficient mice. Flow cytometry analyses show that double deficiencies resulted in an increase of neutrophils in the spleen, accompanied with higher release of interleukin-1β in neutrophils prior to EAE onset. The additional deficiency in NCF1 had no added effect on either interleukin-17 or interferon-γ secretion of T cells during the priming phase.

**Conclusions:**

These studies show that NCF1 and NOS2 interact to regulate peptide-induced EAE.

## Background

Polymorphism of *Ncf1* is a major factor associated with autoimmune diseases, most likely through peroxide regulatory effects [1]. The neutrophil cytosol factor 1 (NCF1), also denoted p47^PHOX^, is a subunit of the NOX2 complex that converts oxygen into superoxide anion. Superoxide is converted to the peroxide but can also react with nitric oxide (NO) in an aqueous environment to yield peroxynitrite anion. Superoxide and peroxynitrite play a dual role in cellular and immune responses [2]. We previously showed that a single nucleotide polymorphism in *Ncf1*, resulting in loss-of-function amino acid substitution, led to an increased risk of developing arthritis [3, 4]. Superoxide defect by mutations in *Ncf1* gene was subsequently shown to cause arthritis and lupus in mice [5, 6], and in humans [7–10].

The immunoregulatory roles of NCF1 have also been studied in experimental autoimmune encephalomyelitis (EAE), which is a widely accepted model to study multiple sclerosis (MS). In the rat model of EAE, the *Ncf1* polymorphism leading to a reduction but not deficiency in superoxide production enhanced the disease severity [11, 12]. In mice, a mutation in *Ncf1* gene, leading to a nearly deficient superoxide production by the NOX2 complex, resulted in an enhanced EAE, in a model that was induced by recombinant rat myelin oligodendrocyte glycoprotein (MOG)_1-125_ protein; in contrast, immunization with a mouse MOG_79-96_ peptide led to reduced EAE [5]. Furthermore, a genetic knockout of *Ncf1* has been reported to result in a complete protection of MOG_35-55_ peptide-induced EAE [13]. It has also been shown that the H-2b mice that were deficient in NCF1 and CYBB subunits of the NOX2 complex were partially protected from EAE when induced by the rat MOG_1-125_ protein or mouse MOG_35-55_ peptide [14, 15].

Inducible nitric oxide synthase termed as NOS2 (alias iNOS) is known to release highly reactive molecules NO under inflammatory conditions [16]. Enhanced expression of NOS2 and peroxynitrite production were observed in mice with EAE [17, 18]. The onset of EAE was delayed after treatment with peroxynitrite scavenger uric acid in *SWXJ-14* mice [19], as well as after treatment of EAE with NO synthase (NOS) inhibitor L-NG-nitroarginine methyl ester (L-NAME) in *C57BL/6* mice [18]. EAE was reduced by using NOS inhibitor aminoguanidine (AG) in *SJL* mice [20]. In contrast, it was observed that AG treatment resulted in exacerbation of EAE in *PL/J* mice [21]. Of interest, the administration of AG or L-NAME showed no effect on EAE in rats [22]. Recent studies have investigated the potential pathogenic mechanisms of NOS2, showing a regulatory role of NOS2 in EAE through peroxynitrite to modulate T cell differentiation in periphery, such as by an expansion of interferon-gamma (IFN-γ)-positive cells [23] and the balance between interleukin-17A (IL-17A)-positive cells [24] and FOXP3-positive cells [25] in *Nos2*-deficient *C57BL/6* mice. In short, these studies of EAE have revealed diverse signaling events downstream of NOS2 deficiency and NOS inhibiting, and complex mechanisms could be mouse strain-dependent.

In this study, we conducted animal experiments by using NCF1- and NOS2-deficient mice of *B6* genetic background and the model of MOG_79-96_ peptide-induced EAE [26]. Our results showed that although NCF1 deficiency leads to a reduced EAE in NOS2-sufficient mice, NCF1 and NOS2 double-deficient mice displayed an enhanced EAE in comparison with NOS2-deficient mice. Flow cytometry analysis of the spleen and lymph node cells shows the innate and adaptive immune responses to immunization. We found an increased number of neutrophils with enhanced IL-1β releases in double-deficient mice following immunization with MOG_79-96_ peptides. Our data point to a possible mechanistic role conferred by NCF1 and NOS2 in enhancing the number of neutrophils that are available to protect against peptide-induced EAE.

## Materials and methods

### Animals

Founders of *B6NQ* (*C57/B6N.Q/rhd*) mice have been fully backcrossed and maintained by Holmdahl laboratory (rhd). A mutation in the *Ncf1* gene (m1j) in the *B6NQ* mice, designated as *B6NQ.Ncf1*^m1j/m1j^, impairs the expression of the *Ncf1* gene, thereby totally blocking the function of the NOX2 complex. NOS2-deficient mice (*B6.129P2-NOS2*^tm1Lau/J^) were obtained from The Jackson Laboratory and were crossed to our *B6NQ* mice to get *C57BL/6NJ.Q.Nos2*^−/−^ mice (*Ncf1*^+/+^.*Nos2*^−/−^) with control *Ncf1*^+/+^.*Nos2*^+/+^ littermates in the experiments here. *Ncf1*^+/+^.*Nos2*^−/−^ mice were crossed with *B6NQ.Ncf1*^m1j/m1j^ mice to generate the C57BL/6NJ.Q.Ncf1^*/*^.*Nos2*^−/−^ mice (Ncf1^*/*^.*Nos2*^−/−^) with control Ncf1^*/*^.*Nos2*^+/+^ littermates. The *Ncf1*^+/*^.*Nos2*^−/−^ mice with a heterogeneous *Ncf1* gene were intercrossed to generate Ncf1^*/*^.*Nos2*^−/−^ mice with control *Ncf1*^+/+^.*Nos2*^−/−^ littermates. Screening for *Ncf1* was performed by TaqMan real-time PCR [27]. The primers for *Nos2* genotyping are as follows: 5′- ACA TGC AGA ATG AGT ACC GG-3′ (common), 5′- TCA ACA TCT CCT GGT GGA AC-3′ (wild type), 5′- AAT ATG CGA AGT GGA CCT CG-3′ (mutant) [28]. All mice in this study expressed the MHC H2-Aq haplotype. Littermate male mice were used in our experiments, and the identity was blinded for the investigator. Mice were housed under specific pathogen-free conditions in individual ventilated cages with wood shaving bedding, a paper napkin as enrichment, and in a climate-controlled environment having a 12-h light/dark cycle. We have mixed experimental cages of 8- to 9-week-old homozygous littermates. Each adult mouse weighed approximately 25 g. Experimental groups were randomized and distributed among mixed cages. The animal study protocols were approved by the Stockholm regional animal ethics committee, Sweden (N83/13).

### Antibodies

The following antibodies were purchased from BioLegend, as CD45 (clone: 30-F11, PerCP/Cy5.5 or PE-Cyanine7), CD11b (clone: M1/70, Pacific Blue), Ly6G (clone: 1A8, PerCP/Cy5.5), Ly-6C (clone: HK1.4, APC or Brilliant Violet 605TM), TNF-α (clone: MP6-XT22, PE-Cyanine7), and IL-17A (clone: TC11-18H10.1, FITC, or APC). Antibodies for CD16/CD32 (clone: 2.4G2, purified), CD3ε (clone: 145-2C11, PerCP/Cy5.5, or PE-Cyanine7), CD4 (clone: RM4-5, Pacific Blue, or PE), and IFN-γ (clone: XMG1.2, APC) were purchased from BD Pharmingen. Antibodies for IL-1β (clone: 166931, FITC) were purchased from R&D Systems. Antibodies for NCF1 (clone: D-10, FITC) were purchased from Santa Cruz Biotechnology. Antibodies for NOS2 (clone: CXNFT, PE-Cyanine7) were purchased from eBioscience. The usage of antibodies is according to the suggestions from the source companies, and the classical dilution ratio of the stock solution is 1:200 for flow cytometry staining.

### Induction and evaluation of EAE

The mice were age-matched and immunized at the base of the tail with 25 μg recombinant MOG_79-96_ peptides emulsified in Freund’s complete adjuvant (CFA, BD Difco, Catalog No. 263810, Sweden). Three hundred nanograms of pertussis toxin from *Bordetella pertussis* (Sigma-Aldrich Co., Catalog No. P2980-.2MG, Sweden) in 100 μL of phosphate-buffered saline (PBS, ThermoFisher, Cat. No. 14190-169, Sweden) was intravenously administrated at the day of immunization and 48 h later. MOG_79-96_ peptide corresponds to amino acids of the mouse sequence (GKVTLRIQNVRFSDEGGY) and was synthesized by Shafer-N, Copenhagen, with a purity of > 97%. The mice will not develop peptide-induced EAE without injection of pertussis toxin, according to the protocol. Clinical signs of EAE were assessed by using a standard scoring protocol [5, 26]. Disease progression was evaluated blindly by the same observer using a clinical scoring as follows: 0, normal; 1, tail weakness; 2, tail paralysis, normal gait; 2.5, tail paralysis, little affected gait; 3, tail paralysis, low back, and mild waddle; 3.5, tail paralysis and low back, severe waddle; 4, tail paralysis, severe waddle, less sure footing; 4.5, tail paralysis, severe waddle, falling and lost balance; 5, tail paralysis and paralysis of one limb, crawling; 6, tail paralysis and paralysis of a pair of limbs, back is affected; and 7, tetra-paresis; 8, pre-morbid or deceased. The endpoint of the experiment is when the mice reach the EAE score of 7. According to the clinical scoring protocol, the onset day is defined as the first day the mouse has shown the clinical symptom with a positive score.

### T cell recall assay

At the time point indicated in the text and figures, the mouse with EAE was euthanized. Detailed time points for the use of CO_2_ euthanasia were day 10 to collect inguinal lymph node cells and day 14 to collect splenocytes post-immunization, respectively. Suspensions of single cells were used for ex vivo analysis. Cells were cultured with MOG_79-96_ peptides (50 μg/mL) for 24 h or 96 h, and then the culture supernatant was collected to determine the level of cytokines and nitric oxide production. The concanavalin A (ConA, Sigma-Aldrich Co., CAS No. 11028-71-0, Sweden) (3 μg/mL) was used as the positive control during the recall assay.

### Nitrite/nitrate detection in medium

The obtained cell culture supernatant samples were stored at − 80 °C until analysis. A commercial nitric oxide (NO_2_/NO_3_) research kit (Enzo Life Sciences, Inc., Catalog No. ADI-917-010, Sweden) was used to determine the level of nitric oxide in a microplate reader (Synergy 2; BioTek, Inc., VT, USA).

### L-NAME treatment

Age-matched mice were administered intraperitoneally with 100 μL volume of NG-nitro-L-arginine methyl ester (L-NAME) (Sigma-Aldrich Co., CAS No. 51298-62-5, Sweden) or PBS once per day for 19 or 20 days after immunization. L-NAME was dissolved into PBS. The dose of L-NAME was 4.3 mg/100 μL/mouse/time, or 172 mg/kg body weight per time, pH 7.4.

### Cytometric beads array

Cytokines levels in the splenic cell culture supernatant were measured by flow cytometry using BD cytometric bead array (CBA, BD Biosciences, Catalog No. 552364, Sweden) mouse soluble protein master buffer kit (IL-1α, GM-CSF, and TNF-α) according to the manufacturer’s instruction. Briefly, 1 × 10^6^ spleen cells were collected from immunized mice, which were re-stimulated with MOG_79-96_ peptides (50 μg/mL) for 24 h at 37 °C.

### Flow cytometry

Flow cytometry was performed on single-cell suspensions from lymph nodes and spleens. The cell density was counted by using Sysmex KX-21 N automated hematology analyzer (Sysmex Corporation, NY). The cell sample was stained with a LIVE/DEAD® fixable near-IR dead cell stain (ThermoFisher, Catalog No. L10119, Sweden). After an anti-mouse CD16/CD32 Fc block, extracellular antigens were stained 20 min at 4 °C in PBS with 1% fetal bovine serum (FBS, Gibco, ThermoFisher, Catalog No. 26140079, USA). To measure intracellular ROS/RNS, the staining of 3 μM Dihydrorhodamine 123 (DHR 123, ThermoFisher, Catalog No. D23806, Sweden), or 5 μM 6-carboxy-2′,7′-dichlorodihydrofluorescein diacetate (DCF, ThermoFisher, Cat. No. C400, Sweden) was conducted respectively after cell surface markers staining, followed by stimulation of 100 ng/mL of phorbol 12-myristate 13-acetate (PMA, Sigma-Aldrich Co., CAS No. 16561-29-8, Sweden) alone or plus 1 μg/mL of ionomycin (ThermoFisher, Catalog No. I24222, Sweden) for 30 min. To detect the intracellular expression of cytokines, the cells were stimulated with 100 ng/mL of PMA and 1 μg/mL of ionomycin in the presence of 5 μg/mL of brefeldin A (BFA, ThermoFisher, Catalog No. B7450, Sweden) for 4 h at a humidified 37 °C, 5% CO_2_ incubator. The stock solutions of PMA, ionomycin, and BFA were prepared with dimethylsulfoxide (DMSO, Sigma-Aldrich Co., CAS No. 67-68-5, Sweden). For intracellular cytokine staining, cells were fixed and permeabilized using BD cytofix/cytoperm solution (BD Biosciences, Catalog No. 554714, Sweden). The workstation is managed by FACSDiva software version 8.0 (BD Biosciences), and the data were analyzed using the FlowJo software version 10.5.3 (TreeStar, Inc., OR).

### Statistics

Statistical analyses were performed with Graph Prism software, version 8.2.1 (GraphPad Software, San Diego, USA). Unless otherwise stated, Mann-Whitney *U* test was used to compare the means of two groups. All results are shown as mean ± standard error of the mean. *p* value < 0.05 was considered as significant: *p < 0.05, **p < 0.01, ***p < 0.001, and ****p < 0.0001.

## Results

### Enhanced EAE is induced in mice deficient in NCF1 and NOS2

**Fig. 1:**
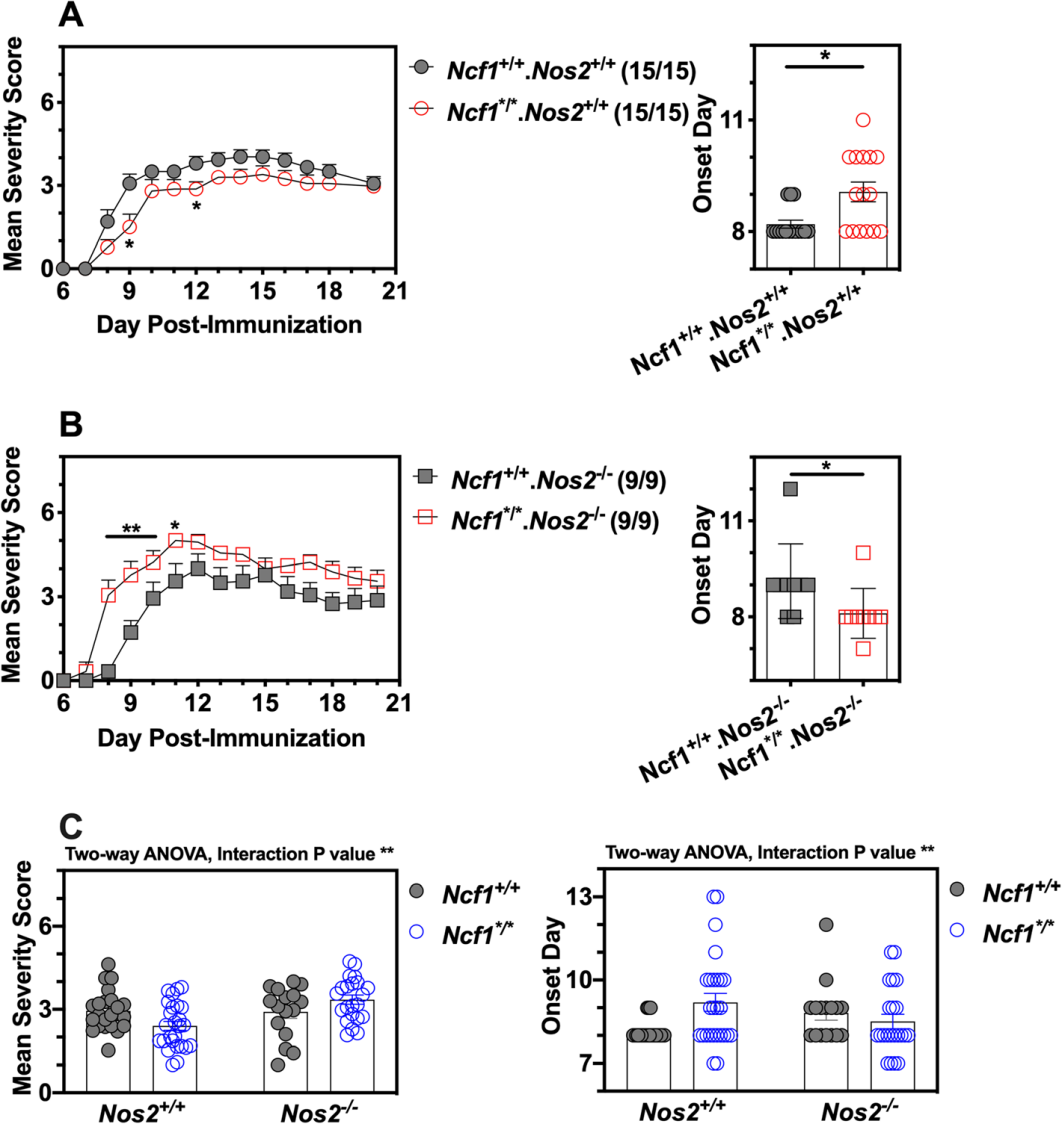
Independent and inter-dependent effects of NCF1 and NOS2 play a dual role in EAE. **a** NCF1 deficiency slightly suppressed the severity and delayed the onset of EAE in mice with normal NOS2. **b** A combined NOS- and NCF1-deficient mice developed an enhanced EAE, together with early onset forms of the disease. In **a** and **b**, the number of mice that developed EAE and the total number of mice in each group are stated in brackets. No mice died before the endpoint. *p < 0.05 and **p < 0.01 as determined in **a** and **b** by the Mann-Whitney *U* test. **c** the interaction *p* values of the two-way ANOVA tables for both mean severity and onset day are less than 0.01, reaching the statistical significance. In **c**, each sign stands for a mouse, and experimental mice of four strains were collected from **a** and **b** and Additional file 1c and 1d

To identify the role of NCF1 and NOS2 in the development of EAE, we have established appropriate mouse strains by crossing *Ncf1*-mutant and *Nos2*-null mice, followed by backcrossing to wild-type (*B6NQ.Ncf1*^+/+^) mice and NCF1-deficient (B6NQ.Ncf1^*/*^) mice. Previous data has shown that NCF1 deficiency can lead to reduced EAE in mice (B10Q.Ncf1^*/*^) if immunized with MOG_79-96_ peptide [5]. In this study, we observed similarly that *Ncf1*-mutant mice (Ncf1^*/*^.Nos2^+/+^) developed milder disease during the early phase of EAE, with a delayed disease onset (Fig. 1a). Based on oxidative burst products generated by the NCF1-NOX2 complex, it is difficult to determine the exact level of peroxynitrite among superoxide, NO, peroxynitrite, and hydrogen peroxide. However, the role of peroxynitrite can be studied by using NOS inhibitor L-NAME [29, 30]. L-NAME treatment results in a delayed onset of EAE (Additional file 1a and 1b). These results suggest that superoxide and peroxynitrite are downstream products of NCF1, promoting inflammation at the initial stage of peptide induced EAE.

We next determined the role of NCF1-derived superoxide in EAE, using NOS2-deficient mice with reduced capacity to form NO and peroxynitrite [29]. Figure 1b shows that double-deficient mice (Ncf1^*/*^.*Nos2*^−/−^) developed EAE with an earlier disease onset and a more severe disease during the early stage than their littermate controls (*Ncf1*^+/+^.*Nos2*^−/−^). Another interesting finding was that NOS2-deficient mice developed a more severe disease during the chronic phase, regardless of NCF1 expression (Additional file 1c and 1d). Based on the data in Fig. 1a and b and Additional file 1c and 1d, the 2-way ANOVA test was performed among wild-type mice, NCF1-deficient mice, NOS2-deficient mice, and NCF1-NOS2-deficient mice. The interaction *p* values of the ANOVA tables for both mean severity and onset day are less than 0.01, reaching the statistical significance (Fig. 1c). Using Bonferroni’s multiple comparisons test of mean severity to determine the significance of the interested pairwise comparison, we found that it is significant from Ncf1^*/*^.*Nos2*^+/+^ mice vs Ncf1^*/*^.*Nos2*^−/−^ mice with a *p* value < 0.001. In Bonferroni’s multiple comparisons test of onset day, it is significant from *Ncf1*^+/+^.*Nos2*^+/+^ mice vs Ncf1^*/*^.*Nos2*^+/+^ mice with a *p* value < 0.05. In summary, these results show a regulatory effect of NCF1 on EAE induction in NOS2-deficient mice (Table 1).

**Table 1:** Descriptive statistics of the disease course across different mouse strains with EAE

Target group	Control group	Acute EAE	Chronic EAE	Data source
Ncf1^*/*^.*Nos2*^+/+^	*Ncf1*^*+*/+^.*Nos2*^+/+^	↓	ns	Fig. 1a
Ncf1^*/*^.*Nos2*^−/−^	*Ncf1*^*+*/+^. *Nos2*^−/−^	↑	ns	Fig. 1b
*Ncf1*^*+*/+^.*Nos2*^−/−^	*Ncf1*^*+*/+^.*Nos2*^+/+^	ns	↑	Additional file 1c
Ncf1^*/*^.*Nos2*^−/−^	Ncf1^*/*^.*Nos2*^+/+^	ns	↑	Additional file 1d
L-NAME+ *Ncf1*^*+*/+^.*Nos2*^+/+^	PBS+ *Ncf1*^*+*/+^.*Nos2*^+/+^	↓	ns	Additional file 1a
L-NAME+ Ncf1^*/*^.*Nos2*^+/+^	PBS+ Ncf1^*/*^.*Nos2*^+/+^	↓	ns	Additional file 1b

Note: The day 14 post-immunization is used to distinguish between acute and chronic EAE. A down arrow stands for a reduced severity of EAE; an up arrow stands for an enhanced severity of EAE; ns stands for no statistical significance. The comparison between two groups was determined by the Mann-Whitney *U* test

Results from in vivo analyses provide evidence that EAE is regulated by NCF1 and NOS and that NCF1 is protective during EAE induction. Additionally, a regulatory role is likely for NOS2 during EAE remission.

### T cell immune response to antigens is not regulated by NCF1 and NOS2 deficiencies

**Fig. 2:**
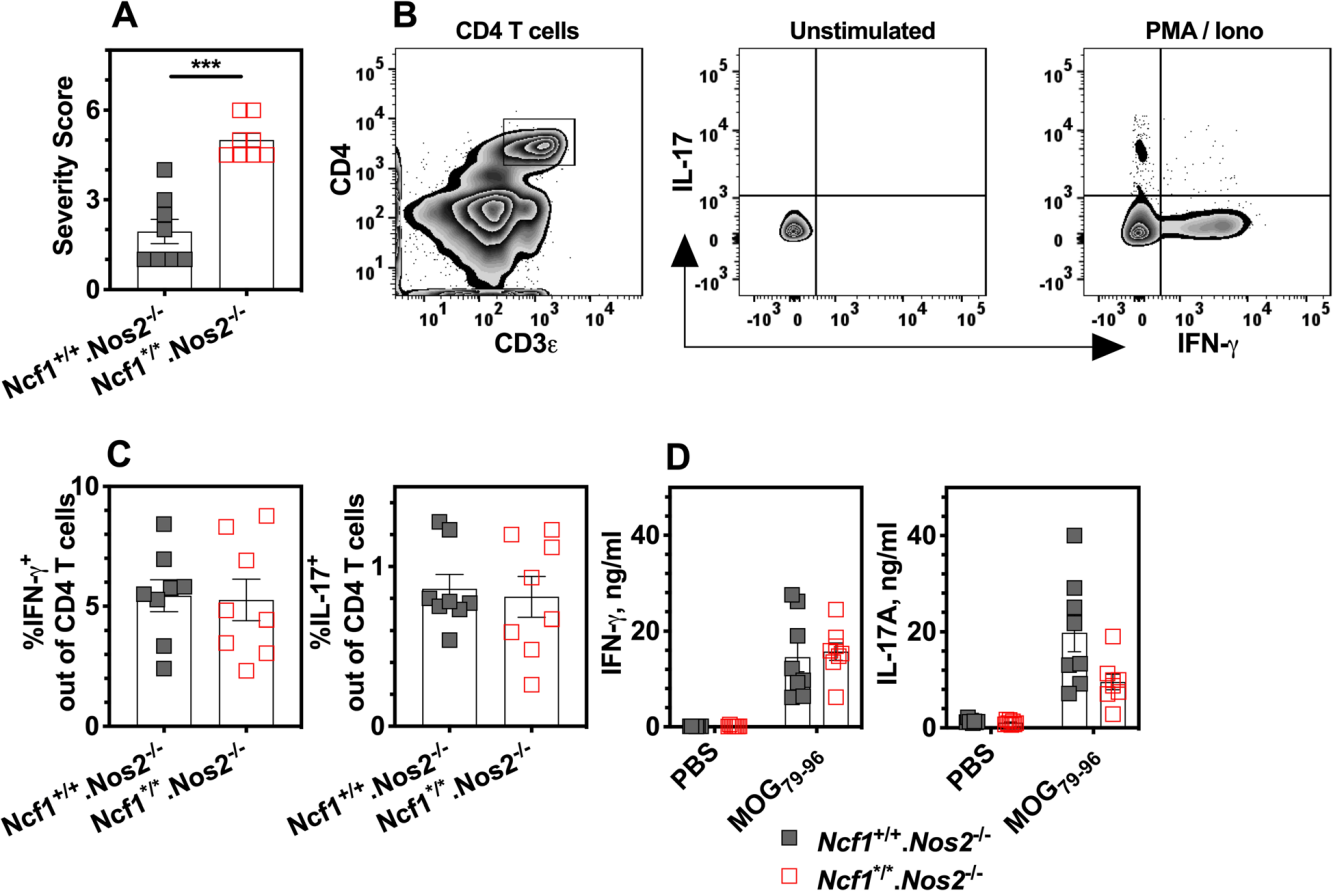
The enhanced EAE is not associated with cytokine productions of T cell subsets. **a** NOS2-deficient mice were immunized with the MOG_79-96_ peptides and sacrificed at day 10 after immunization. The EAE severity was higher in double-deficient mice (Ncf1^*/*^.*Nos2*^−/−^ genotype) than that of their littermate controls (*Ncf1*^+/+^.*Nos2*^−/−^ genotype), of which inguinal lymph node cells were analyzed by using flow cytometric analysis as follows. **b** Representative plots for IFN-γ and IL-17-positive cells in CD4^+^ T cells with or without PMA and ionomycin stimulation. **c** The frequencies of IFN-γ and IL-17-positive cells in CD4^+^ T cells were similar after stimulation with PMA and ionomycin for 4 h. **d** Concentrations of IFN-γ and IL-17 in lymph node cell culture supernatant after re-stimulation ex vivo using MOG_79-96_ peptides for 96 h. The number of mice in each group is 8. ***p < 0.001 as determined by the Mann-Whitney *U* test

To study redox mechanisms of enhanced EAE in double-deficient mice, we firstly examined adaptive immune responses to immunization. The previous study of NOS2 mice showed that inter-dependent regulation of NOX2 and NOS2 in IL-17-positive T cells was critical to enhanced diseases [24, 25]. Therefore, we determined the effect of oxidative burst on T cells characterized by production of IFN-γ and IL-17 during EAE induction. At day 10 post immunization, we collected inguinal lymph nodes for flow cytometric analysis, using double-deficient mice (Ncf1^*/*^.*Nos2*^−/−^) that developed more severe EAE than their littermates (*Ncf1*^+/+^.*Nos2*^−/−^ genotype) (Fig. 2a). We found that frequencies of neither IFN-γ nor IL-17A-positive cells in NOS2-deficient CD4 T cells were influenced by NCF1 deficiency, upon re-stimulation ex vivo with PMA and ionomycin (Fig. 2b, c). In addition, we measured the level of cytokine production in cell cultures after re-stimulation ex vivo with MOG_79-96_ peptides. The levels of IFN-γ and IL-17A after antigen recall stimulation were similar between the two groups from Ncf1^*/*^.*Nos2*^−/−^ mice and *Ncf1*^+/+^.*Nos2*^−/−^ littermates (Fig. 2d).

In comparison with lymph nodes, spleens have a higher frequency of myeloid cells that express both NCF1 and NOS2. Therefore, we performed a recall assay on spleen cells from mice at day 14 post-immunization, when the disease was very severe (Additional file 2a). Although we observed a decrease in NO production from NOS2-deficient spleen cells stimulated ex vivo with ConA (Additional file 2b), there was no difference in IL-17A level between these groups (Additional file 2c and 2d).

In summary, we did not find that NCF1-dependent effect on antigen-specific T cells responses during the early and peak stages of EAE in NOS2 deficient mice.

### EAE induction is associated with oxidative burst of neutrophils

To study redox mechanisms of EAE, we next examined innate immune responses to immunization. We focused on the myeloid subset responsible for oxidative burst prior to EAE clinical onset. A previous study showed that upon PMA stimulation, there was no difference in NCF1 expression and oxidation burst of T cells between naïve NCF1 wild-type (*B10Q.Ncf1*^+/+^) mice and NCF1-deficient (B10Q.Ncf1^*/*^) littermates, and antigen-specific T-cell activation was controlled by oxidative signaling from macrophages [27]. Myeloid cells including neutrophils and monocytes/macrophages, typically expressing both NCF1 and NOS2, were recently shown to be important in the regulation of EAE at the induction phase by releasing IL-1β [31].

**Fig. 3:**
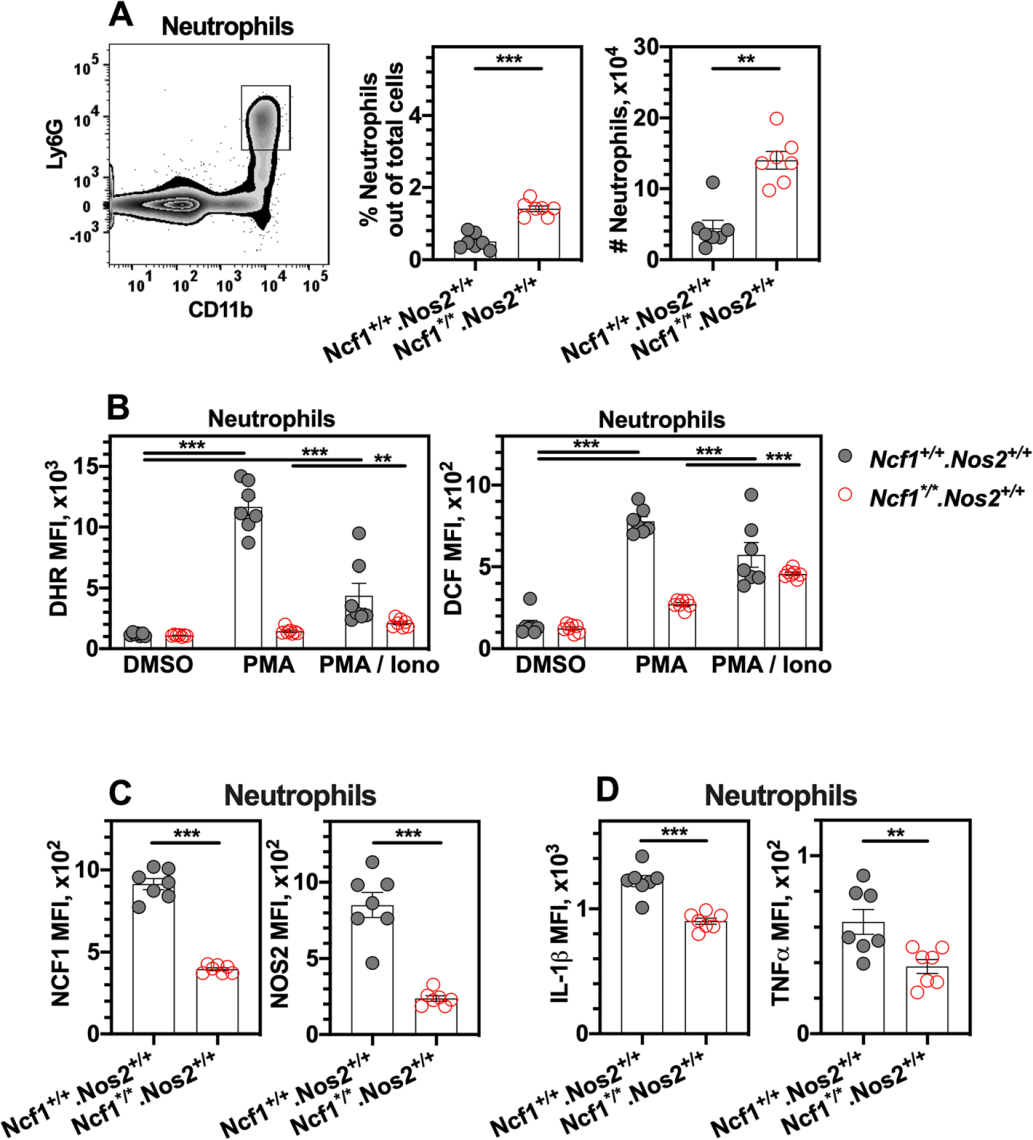
NCF1 deficiency reduces IL-1β release from neutrophils in spleens prior to clinical onset. **a** Representative flow cytometry plot for neutrophils in the spleen. The solenocytes were collected from NCF1-deficient (Ncf1^*/*^.*Nos2*^+/+^ genotype) and NCF1-sufficient mice (*Ncf1*^+/+^*.Nos2*^+/+^ genotype) at day 4 post immunization. The frequency and cell number of neutrophils in the spleen are shown, upon stimulation with PMA. **b** Mean florescence intensities (MFIs) of DHR and DCF staining in neutrophils are shown, after cells were incubated ex vivo with PMA, PMA and ionomycin, or DMSO as the control. **c** MFIs of NCF1 and NOS2 staining in neutrophils are shown. **d** MFIs of IL-1β and TNF-α staining in neutrophils are shown. The number of mice is 7 per group. **p < 0.01 and ***p < 0.001 as determined by the Mann-Whitney *U* test

In the EAE model, we first measured the intracellular oxidative status in splenic myeloid cells and T cells from mice (*Ncf1*^+/+^.*Nos2*^+/+^ and Ncf1^*/*^.*Nos2*^+/+^ genotypes) at day 4 post-immunization, using fluorescent dyes DHR and DCF by flow cytometric analysis [32], as shown in Fig. 3a and Additional file 3 and 4. DHR was used to detect peroxynitrite oxidation, and DCF was used for detecting hydrogen peroxide. Upon re-stimulation ex vivo with PMA, we found that NCF1 deficiency resulted in a higher frequency of splenic neutrophils. We observed that a little or no oxidative burst from NCF1-deficient myeloid cells upon PMA or PMA/ionomycin stimulation (Fig. 3b and Additional file 3), whereas expression of both NCF1 and NOS2 in neutrophils was shown in Fig. 3c as evidence. There was also a very low level of oxidative burst from CD4 T cells, even though an elevated level was detected post stimulation, irrespective of NCF1 expression (Additional file 4). Therefore, it suggests that NCF1-derived oxidative burst is not the main source of oxidative signals in CD4 T cells. Additionally, flow cytometry analyses allow us to determine the level of cytokine production of myeloid cells, such as IL-1β and tumor necrosis factor-α (TNF-α). NCF1 deficiency led to lower levels of pro-IL-1β and TNF-α in neutrophils (Fig. 3d), but not Ly6C^hi^ monocytes (Additional file 3).

**Fig. 4:**
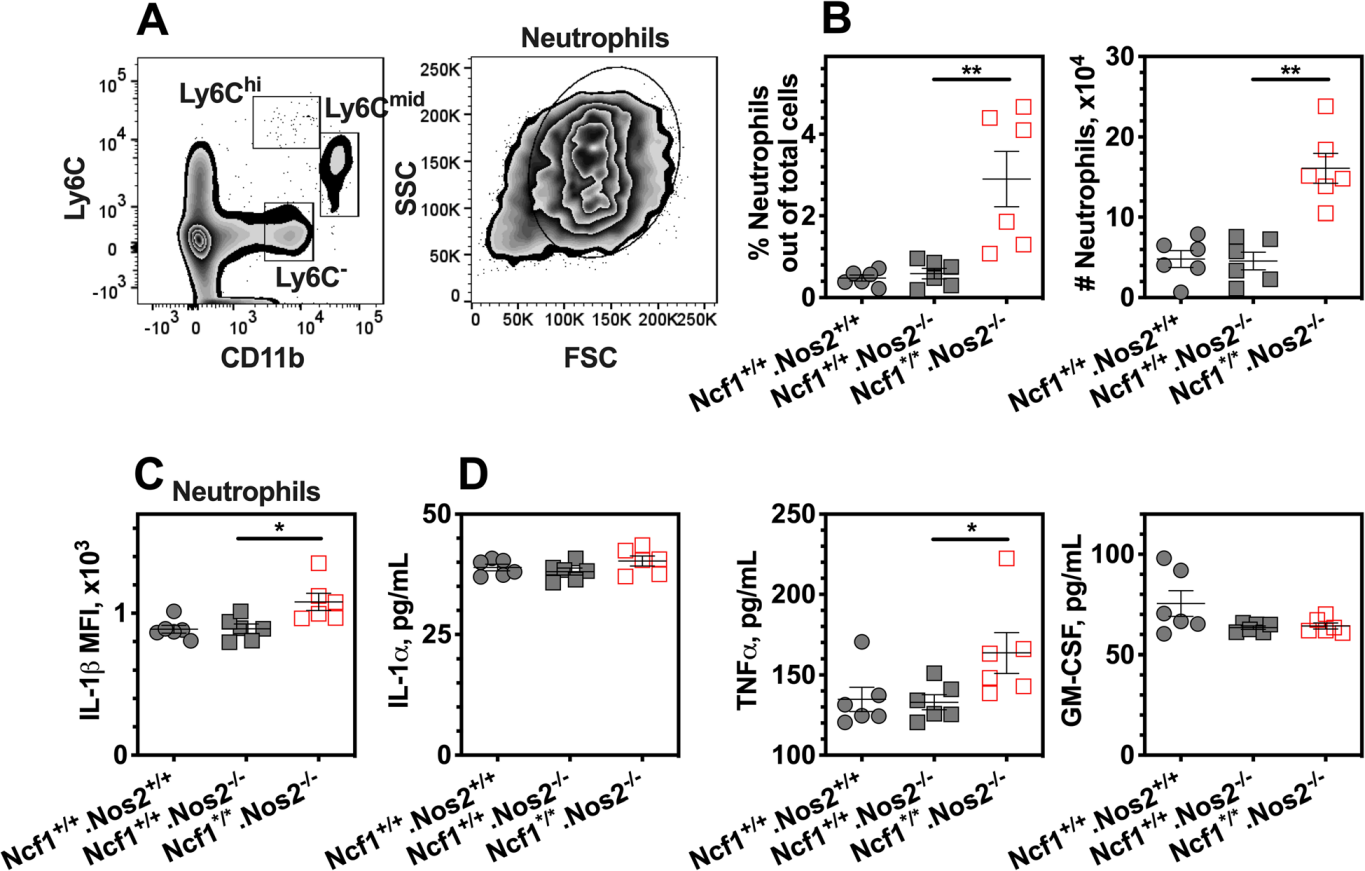
Double deficiencies of NCF1 and NOS2 increase IL-1β release from neutrophils in the spleen prior to clinical onset. **a** Representative flow cytometry plots for neutrophils in the spleen. The splenocytes were collected from double-deficient mice (Ncf1^*/*^.*Nos2*^−/−^ genotype) and their littermates NOS2-deficient mice (*Ncf1*^+/+^.*Nos2*^−/−^ genotype), as well as wild-type mice (*Ncf1*^+/+^*.Nos2*^+/+^ genotype) at day 4 post immunization. **b** The frequency and cell number of neutrophils in the spleen are shown, upon stimulation with PMA. **c** The MFIs of IL-1β staining in splenic neutrophils are shown. **d** The cytokine levels of spleen cell culture supernatants were determined after incubation with MOG_79-96_ peptides for 24 h. The number of mice per group is 6. *p < 0.05 and **p < 0.01 as determined by the Mann-Whitney *U* test

We finally used NCF1 and NOS2 double-deficient mice to further study redox regulation of innate immune responses to immunization. On basis of the above observations of reduced IL-1β and TNF-α in NCF1-deficient neutrophils, we conducted similar experiments with flow cytometry analysis of spleen cells in Ncf1^*/*^.*Nos2*^−/−^, *Ncf1*^+/+^.*Nos2*^−/−^, and wild-type mice (*Ncf1*^+/+^.*Nos2*^+/+^) at day 4 post-immunization. Similar to the previous results in NOS2-sufficient mice (Fig. 3a), NCF1 deficiency led to an increased frequency of neutrophils in the spleen (CD11b^+^Ly6C^mid^, Fig. 4a, b), upon re-stimulation ex vivo with PMA. Importantly, an increased pro-IL-1β expression was shown in NCF1-deficient neutrophils (Fig. 4c), but not found in Ly6C^hi^ monocytes and Ly6C^-^ myeloid cells (Additional file 5). In addition, since GM-CSF-positive T cell response could be regulated by neutrophils, we studied the immune responses against MOG_79-96_ peptides by using a recall antigen assay to understand oxidative signaling. The splenic cells were re-stimulated with MOG_79-96_ peptides for 24 h, and cell culture supernatants were analyzed. Neither IL-1α nor GM-CSF was upregulated in the cell culture supernatants from double-deficient cells. Nevertheless, double deficiencies in NCF1 and NOS2 resulted in an increased TNF-α production in the recall antigen assay (Fig. 4d).

In summary, we conclude that the number of neutrophils in the spleen and their IL-1β secretions are most closely associated with oxidative signaling in control of EAE induction.

## Discussion

The oxidative burst derived by the NCF1-NOX2 complex could play a dual role in autoimmune disease. In NOS2-sufficient mice, we show that NCF1-dependent oxidative burst played a pathogenic role during peptide-induced EAE; however, based on NOS2-deficient mice, we find that NCF1-dependent oxidative burst was protective in EAE induction. The inter-dependent effects of NCF1 and NOS2 were associated with neutrophils rather than T cells in immune responses to immunization.

A promising concept to explain differences in EAE susceptibility is the regulation of IL-1β release by neutrophil-derived oxidative burst. The oxidative mechanisms underlaying transcription of the gene encoding the IL-1β precursor pro-IL-1β, pro-IL-1β processing, and IL-1β cellular export are not understood well yet. IL-1β transcription can be induced by pertussis toxin at the time of immunization, and increased recruitment of pro-IL-1β-producing neutrophils from the bone marrow to draining lymph nodes and the spleen was required for EAE induction [33–35]. Interestingly, the peak number of blood-derived neutrophils infiltrated into the brain of *C57/BL6* mice was on day 4 post injection of pertussis toxin alone [33]. Neutrophil depletion using the 1A8 monoclonal antibody delayed the onset of EAE and attenuated clinical symptoms [36], and a similar effect was observed in *C57/BL6* mice deficient in inflammasome genes (e.g., *caspase-1*^*−/−*^and *GsdmD*^*−/−*^) involved in the processing of pro-IL-1β to active secreted IL-1β [37, 38]. *IL-1β*^*−/−*^mice displayed the reduced EAE and also failed to remyelinate properly involved with the function of microglia or macrophages [39] and NOS2 [40]. In addition, NOX2-deficient mice exhibited an attenuation of EAE-induced IL-1β transcription in the brain at the day 20 post-immunization [41]. In this paper, we furthered the studies of neutrophil-dependent regulation of EAE development in the mouse model. We observed that NCF1 deficiency resulted in an increased number of neutrophils in the spleen. NCF1-deficient neutrophils produced less both IL-1β and TNF-α, associated with delayed disease onset and reduced severity of EAE. Of interest, our results show that double-deficient mice in NCF1 and NOS2 exhibited an increase pro-IL-1β expression in neutrophils and a rapid development of enhanced EAE. Moreover, we did not find a similar increase in monocyte-limited pro-IL-1β expression in double-deficient mice, and our results are different from the published data that the periphery monocyte was the key to drive EAE by releasing IL-1β [42].

A possible explanation could be that the cytotoxicity of oxidative burst via peroxynitrite could modulate IL-17-production of CD4 T cells. It is a classical phenotype that RORγt and IL-17-expressing CD4 T cells transfer EAE to the naïve hosts [43]. A molecular mechanism for encephalitogenic IL-17 production is the intrinsic post-translational modification of RORγt by peroxynitrite [24]. The T cell endogenous peroxynitrite is a natural product at the interaction between NCF1 and NOS2 [24]. Peroxynitrite can be generated by surrounding cells in short-range and long-range mechanisms. By using an autocrine manner, T cells could produce superoxide likely by the NCF1-NOX2 complex [44, 45] and mitochondria [46] and NO by the NOS. Antigen-specific T cells can be also exposed to oxidative burst provided by monocytes/macrophages [27, 47] and endothelial cells [48] in a paracrine manner. In this study, we observed that the MFI of DHR staining in CD4 T cells was near to the background value, which was 10 times less than in splenic neutrophils and monocytes from the mice after immunization with MOG_79-96_ peptides. Although we showed a weak production of superoxide and H_2_O_2_ in CD4 T cells induced by ionomycin together with PMA, it was clearly independent of NOX2 activity as evidenced by a similar MFI of DCF staining in NCF1 deficient cells. Moreover, we found little or no measurable expression of NCF1 and NOS2 in CD4 T cells from MOG_79-96_ peptides immunized mice. We detected a similar level of IL-17 in primed CD4 T cells from NOS2-deficient mice, regardless of NCF1 expression. Therefore, our results suggest that the neutrophil-dependent mechanism underlying enhanced EAE in double-deficient mice should be different from peroxynitrite-IL-17 producing T cell pathways.

Other mechanisms regulated the EAE induction could be TNF-α and GM-CSF pathways mediated by neutrophils. A GM-CSF-dependent signaling has shown that IFN-γ-deficient T cells can induce neutrophil-rich infiltrates and transfer EAE to the naïve host irrespective IL-17 signaling [49], and it has also been recently shown dispensable for disease induction [50]. TNF-deficient mice showed reduced EAE during the early stage but exacerbated disease during the chronic stage due to prolonged retention of T cells in the secondary lymphoid organs [51]. TNF-α ablation in monocytes/macrophages delayed the onset of EAE in challenged animals [52]. An interesting evidence by studies with humanized mice indicates that the soluble TNF-α signaling provided a protective effect on EAE induction through TNFR2 on the CD4^+^FOXP3^+^ cells in the spleen, but not T cells in the CNS [53]. Our earlier data also showed that CTLA-4 deletion in adult mice resulted in an increase of CD4^+^FOXP3^+^ cells in the spleen and lymph nodes, leading to resistance to MOG_79-96_ peptide-induced EAE [54]. In the present study, we did not observe any difference on protein expression of GM-CSF from mice deficient in NCF1 and NOS2 during EAE induction, using a recall antigen assay of splenic and lymph node cells. Our data shows that NCF1 and NOS2 deficiencies resulted in an increase of TNF-α production, compared with the wild-type or NOS2-deficient group. Therefore, oxidative regulation will be an interesting follow-up study by assaying the function of TNF-α-TNFR2 signaling on the CD4^+^FOXP3^+^ cells.

## Conclusion

**Fig. 5:**
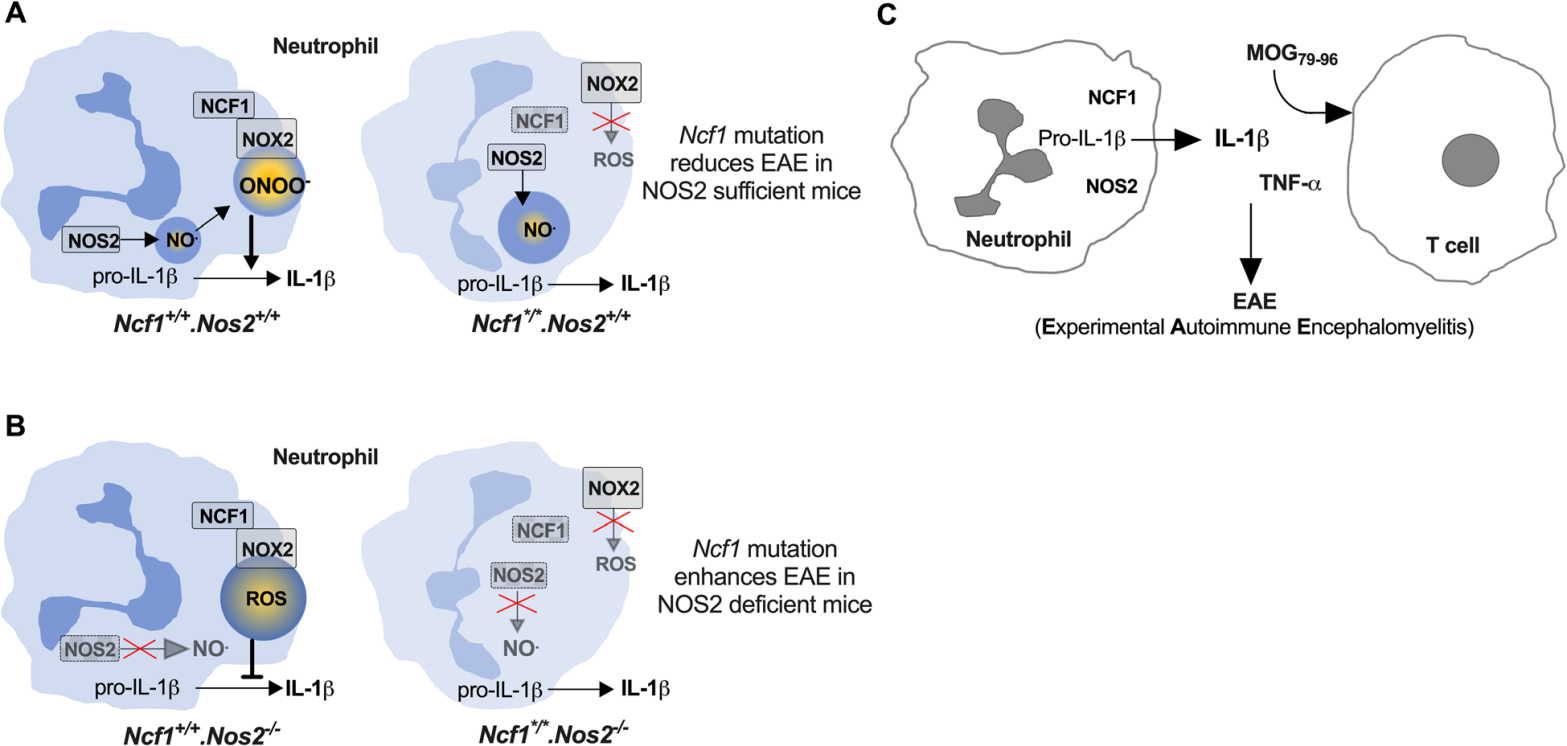
Schematic graph illustrating the association of both *Ncf1* and *Nos2* with MOG peptide-induced EAE. The *Ncf1*-mediated regulation was associated with IL-1β secreted by neutrophils in the response to pertussis toxin in mice during the early stage of EAE development. **a***Ncf1* mutation impaired ROS production by the NOX2 complex, decreased IL-1β production by neutrophils, and reduced EAE in *Nos2*-sufficient mice. **b***Ncf1* mutation enhanced IL-1β production by neutrophils and increased EAE in *Nos2*-deficient mice that failed to produce nitric oxide. NOS2 deficiency enhanced EAE in the chronic phase, irrespective of *Ncf1* status. **c** TNF-α production was increased in response to MOG_79-96_ stimulation in *Ncf1*-*Nos2*-deficient mice after immunization. In summary, a potential mechanism underlying the interaction between neutrophils and T cells might regulate the EAE development in an NCF1-NOS2-dependent manner

In conclusion, this study demonstrates that NCF1 and NOS2 can operate as a stand-alone or inter-dependent regulator, showing a dual role in the EAE model. The pathogenic effects of NCF1 on EAE induction were verified in NCF1-deficient mice and in wild-type mice with L-NAME treatments. The protective effect of NCF1 on EAE was shown in NOS2-deficient mice. NCF1-NOS2 double deficiency led to an increased number of neutrophils in the spleen and a higher release of IL-1β by neutrophils prior to clinical onset, followed by an exacerbated EAE. In addition, although NCF1 deficiency did not provide an added effect on IFN-γ or IL-17 production of antigen-specific T cells during the early stage of EAE in NOS2-deficient mice, the double deficiencies resulted in an increased TNF-α production in the antigen recall assay. A potential mechanism underlying the interaction between neutrophils and T cells might regulate the EAE development in an NCF1-NOS2 dependent manner (Fig. 5).

## Supplementary information

**Additional file 1: **tiff format, title: NCF1 and NOS2 play a dual role in EAE. **a**, treatments of NOS inhibitor L-NAME suppress the induction of EAE in wild type mice (*Ncf1*^+/+^.*Nos2*^+/+^ genotype). **b**, treatments of L-NAME suppress the induction of EAE in NCF1-deficient mice (Ncf1^*/*^.*Nos2*^+/+^ genotype), but prevent the chronic remission. Weight restoration was a crucial component of EAE remission. In the absence of NCF1, L-NAME treatment led to a failure at weight restoration at the chronic stage. **c**, a prolonged EAE is shown due to NOS2 deficiency in wild type mice, accompanying with the failure of weight restoration after day 15 post immunization. **d**, NOS2 deficiency enhances EAE in NCF1 deficient mice. The number of mice that developed EAE and the total number of mice in each group are stated in brackets. *p < 0.05, **p < 0.01, ***p < 0.001 and ****p < 0.0001 as determined by the Mann-Whitney U test.

**Additional file 2: **tiff format, title: Double deficiencies do not result in any difference on IL-17 production in a recall assay of spleen cells, compared with NOS2 deficiency. **a**, before euthanasia, clinical scores of EAE were evaluated at day 14 post-immunization, among *Ncf1*^+/+^.*Nos2*^+/+^ mice and their Ncf1^*/*^.*Nos2*^+/+^ littermates, together with *Ncf1*^+/+^.*Nos2*^-/-^ mice and their Ncf1^*/*^.*Nos2*^-/-^ littermates. Spleens were isolated and used in the re-stimulation assay *ex vivo*. **b**, the level of nitrite plus nitrate is measured as an indicator of NO production and, **c**, IL-17 concentration in the supernatant is characterized as a positive control in the T cell assay after stimulation using ConA for 96 h. **d**, IL-17 production in the supernatant was measured in the recall assay using MOG_79-96_ peptides. The number of mice that developed EAE and the total number of mice are stated in brackets. *p < 0.05 and **p < 0.01 as determined by the Mann-Whitney U test.

**Additional file 3: **tiff format, title: NCF1 deficiency has no effect on IL-1β release from Ly6C^hi^ monocytes in the spleen prior to clinical onset. **a**, a representative flow cytometry plot for Ly6C^hi^ monocytes in the spleen. The splenocytes were collected from NCF1 deficient (Ncf1^*/*^.*Nos2*^+/+^) and sufficient mice (*Ncf1*^+/+^*.Nos2*^+/+^) at day 4 post immunization. The frequency and cell number of Ly6C^hi^ monocytes in the spleen are shown, upon stimulation with PMA. **b**, mean florescence intensities (MFIs) of DHR and DCF staining of Ly6C^hi^ monocytes are shown, after these cells were incubated *ex vivo* with PMA, PMA and ionomycin or DMSO as the control. **c**, the MFIs of IL-1β and TNF-α staining in Ly6C^hi^ monocytes are shown. The number of mice is 7 per group. **p < 0.01 and ***p < 0.001 as determined by the Mann-Whitney U test.

**Additional file 4: **tiff format, title: There is little or no detectable NCF1 and NOS2 expression in CD4 T cells in the spleen prior to clinical onset. **a**, a representative flow cytometry plot for CD4 T cells in the spleen. The splenocytes were collected from NCF1 deficient (Ncf1^*/*^.*Nos2*^+/+^) and sufficient mice (*Ncf1*^+/+^*.Nos2*^+/+^) at day 4 post immunization. The frequency and cell number of CD4 T cells in the spleen are shown, upon stimulation with PMA. **b**, mean florescence intensities (MFIs) of DHR and DCF staining of CD4 T cells are shown, after these cells were incubated *ex vivo* with PMA, PMA and ionomycin or DMSO as the control. **c**, the MFIs of NCF1 and NOS2 staining in CD4 T cells are shown. The number of mice is 7 per group. **p < 0.01 and ***p < 0.001 as determined by the Mann-Whitney U test.

**Additional file 5: **tiff format, title: There is no detectable change of pro-IL-1β expression in Ly6C^hi^ monocytes and Ly6C^-^ myeloid cells in the spleen prior to clinical onset. **a**, here are the frequencies of Ly6C^hi^ monocytes and Ly6C^-^ myeloid cells stated in Fig. 4a, upon stimulation with PMA. **b**, the MFIs of IL-1β in selected subsets are shown. The number of mice per group is 6. **p < 0.01 as determined by the Mann-Whitney U test.

## Data Availability

All data generated or analyzed during this study are included in this published article [and its supplementary information files].

## Abbreviations

DCF: 6-Carboxy-2′,7′-dichlorodihydrofluorescein diacetate
DHR: Dihydrorhodamine 123
EAE: Experimental autoimmune encephalomyelitis
GM-CSF: Granulocyte-macrophage colony-stimulating factor
IFN-γ: Interferon-gamma
IL-1β: Interleukin 1β
IL-17: Interleukin 17A
L-NAME: NG-nitro-L-arginine methyl ester
NCF1: Neutrophil cytosol factor 1
NO: Nitric oxide
NOS: Nitric oxide synthase
NOS2: iNOS, inducible nitric oxide synthase
MOG: Myelin oligodendrocyte glycoprotein
MS: Multiple sclerosis
PMA: Phorbol 12-myristate 13-acetate
TNF-α: Tumor necrosis factor-α
