# Supplementary figures and images for: Independent and inter-dependent immunoregulatory effects of NCF1 and NOS2 in experimental autoimmune encephalomyelitis
Source: J Neuroinflammation. 2020 Apr 11;17:113. doi: 10.1186/s12974-020-01789-2 (PMC7149911; doi:10.1186/s12974-020-01789-2)

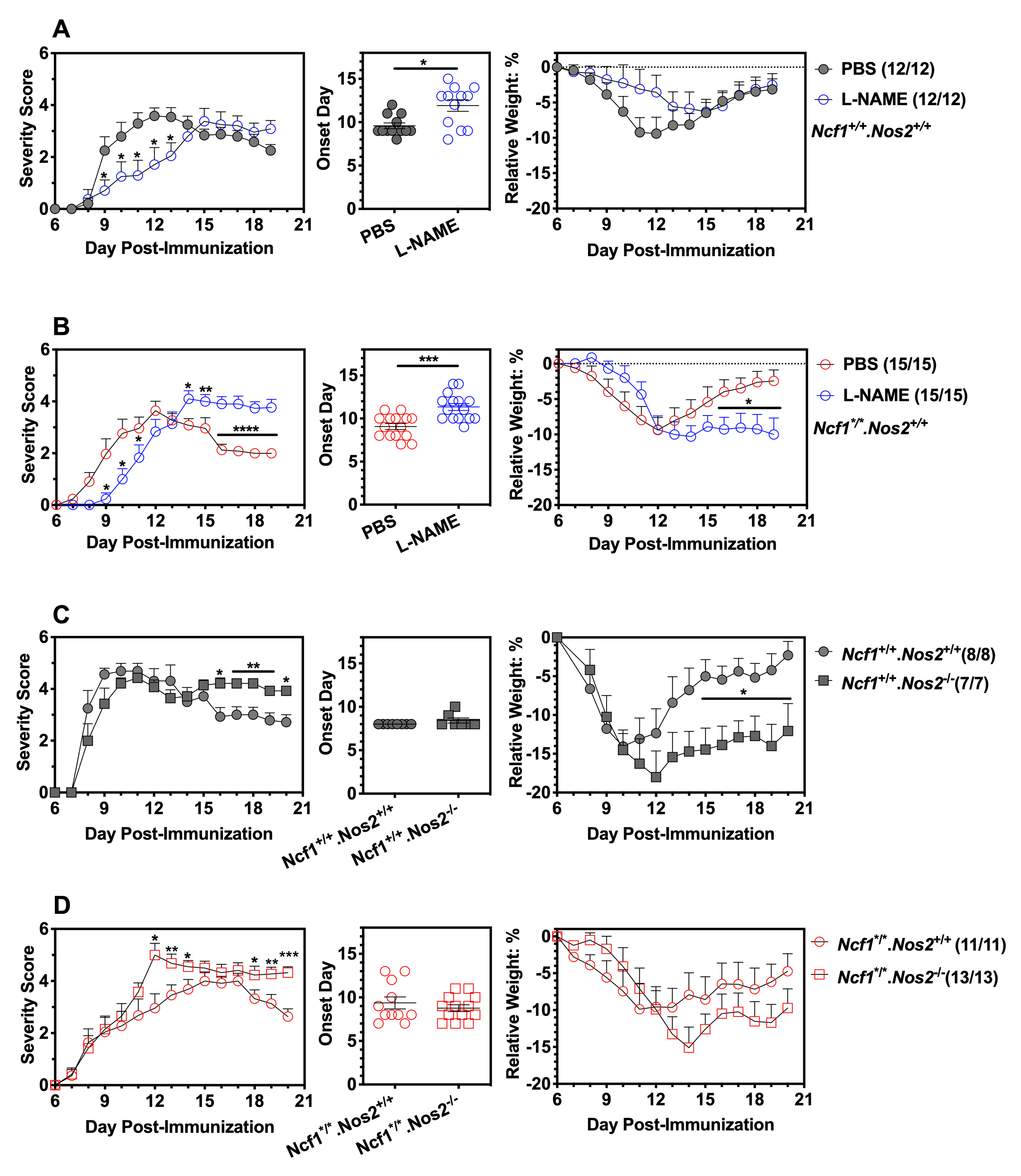

Supplement: Supplementary file 1 — Additional file 1: tiff format, title: NCF1 and NOS2 play a dual role in EAE. a, treatments of NOS inhibitor L-NAME suppress the induction of EAE in wild type mice (Ncf1+/+.Nos2+/+ genotype). b, treatments of L-NAME suppress the induction of EAE in NCF1-deficient mice (Ncf1*/*.Nos2+/+ genotype), but prevent the chronic remission. Weight restoration was a crucial component of EAE remission. In the absence of NCF1, L-NAME treatment led to a failure at weight restoration at the chronic stage. c, a prolonged EAE is shown due to NOS2 deficiency in wild type mice, accompanying with the failure of weight restoration after day 15 post immunization. d, NOS2 deficiency enhances EAE in NCF1 deficient mice. The number of mice that developed EAE and the total number of mice in each group are stated in brackets. *p < 0.05, **p < 0.01, ***p < 0.001 and ****p < 0.0001 as determined by the Mann-Whitney U test. [file 12974_2020_1789_MOESM1_ESM.tiff]

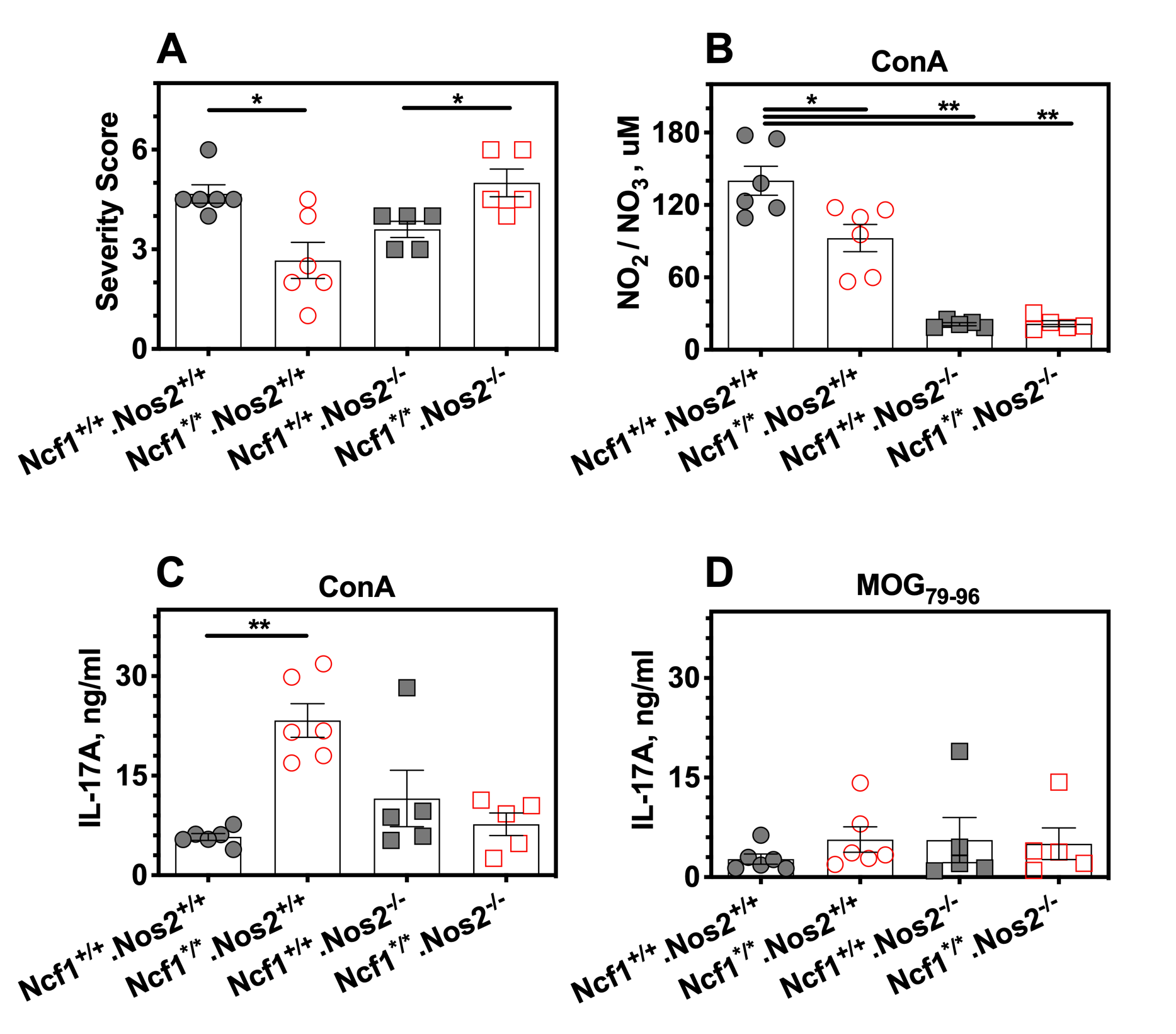

Supplement: Supplementary file 2 — Additional file 2: tiff format, title: Double deficiencies do not result in any difference on IL-17 production in a recall assay of spleen cells, compared with NOS2 deficiency. a, before euthanasia, clinical scores of EAE were evaluated at day 14 post-immunization, among Ncf1+/+.Nos2+/+ mice and their Ncf1*/*.Nos2+/+ littermates, together with Ncf1+/+.Nos2-/- mice and their Ncf1*/*.Nos2-/- littermates. Spleens were isolated and used in the re-stimulation assay ex vivo. b, the level of nitrite plus nitrate is measured as an indicator of NO production and, c, IL-17 concentration in the supernatant is characterized as a positive control in the T cell assay after stimulation using ConA for 96 h. d, IL-17 production in the supernatant was measured in the recall assay using MOG79-96 peptides. The number of mice that developed EAE and the total number of mice are stated in brackets. *p < 0.05 and **p < 0.01 as determined by the Mann-Whitney U test. [file 12974_2020_1789_MOESM2_ESM.tiff]

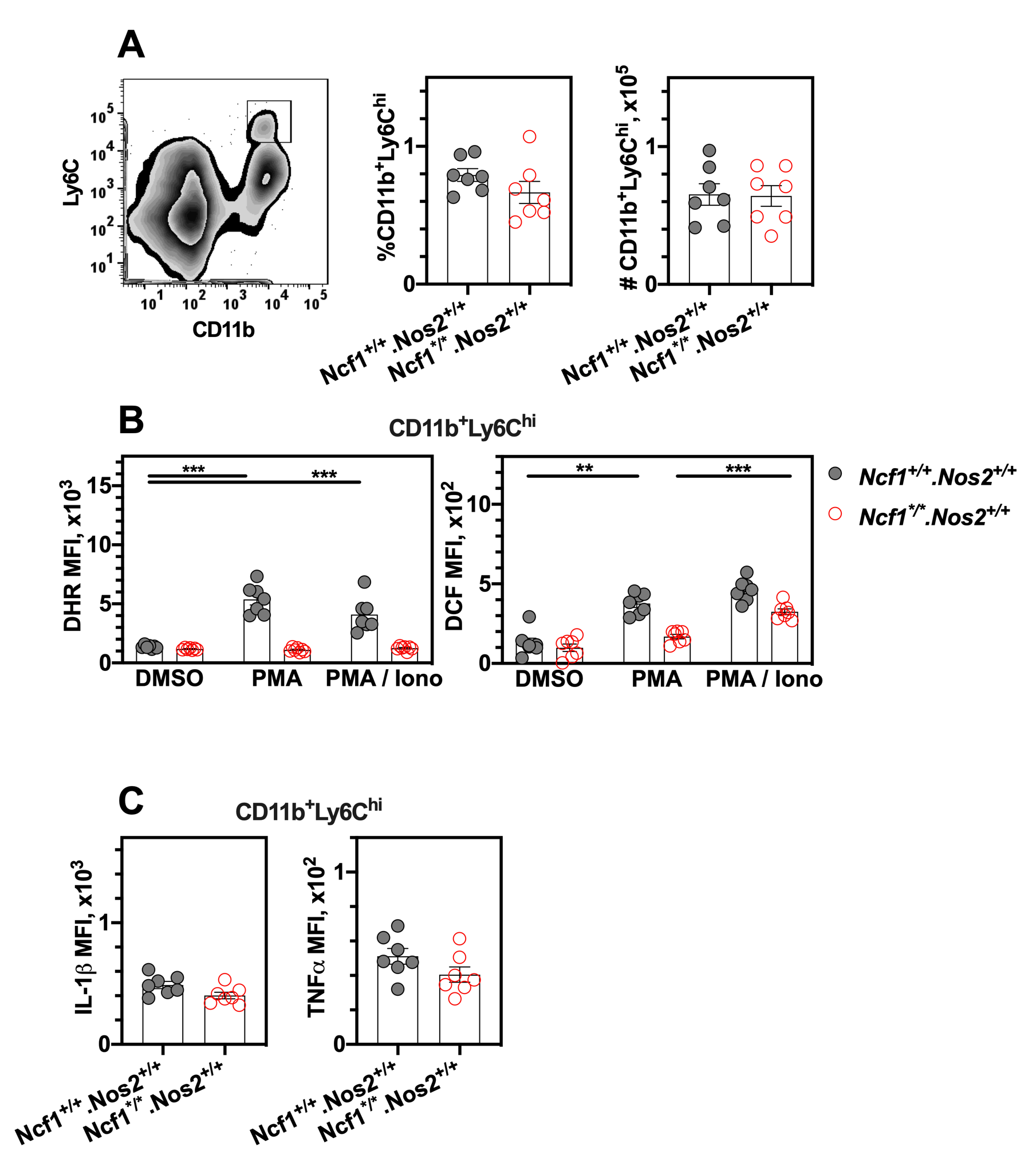

Supplement: Supplementary file 3 — Additional file 3: tiff format, title: NCF1 deficiency has no effect on IL-1β release from Ly6Chi monocytes in the spleen prior to clinical onset. a, a representative flow cytometry plot for Ly6Chi monocytes in the spleen. The splenocytes were collected from NCF1 deficient (Ncf1*/*.Nos2+/+) and sufficient mice (Ncf1+/+.Nos2+/+) at day 4 post immunization. The frequency and cell number of Ly6Chi monocytes in the spleen are shown, upon stimulation with PMA. b, mean florescence intensities (MFIs) of DHR and DCF staining of Ly6Chi monocytes are shown, after these cells were incubated ex vivo with PMA, PMA and ionomycin or DMSO as the control. c, the MFIs of IL-1β and TNF-α staining in Ly6Chi monocytes are shown. The number of mice is 7 per group. **p < 0.01 and ***p < 0.001 as determined by the Mann-Whitney U test. [file 12974_2020_1789_MOESM3_ESM.tiff]

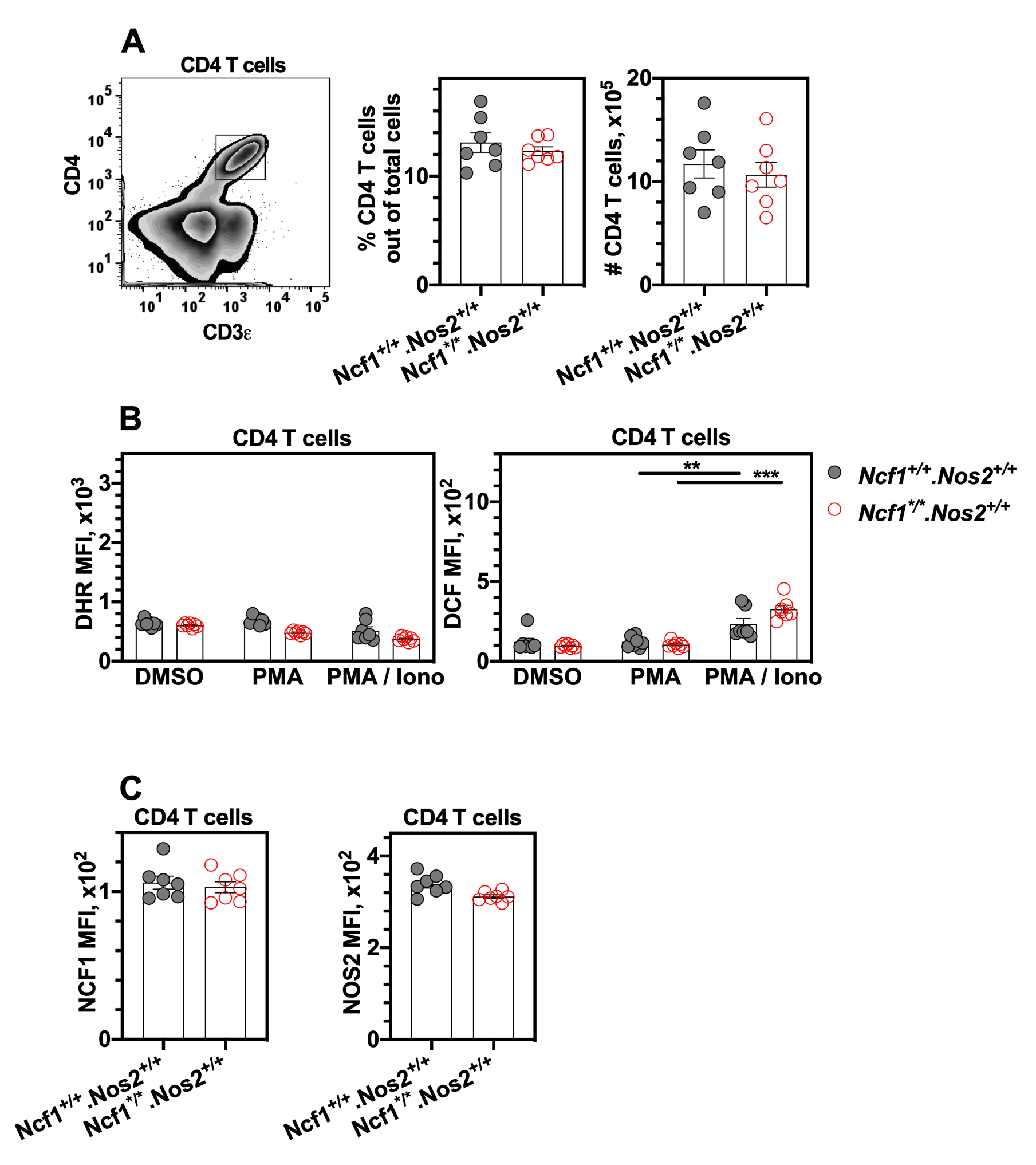

Supplement: Supplementary file 4 — Additional file 4: tiff format, title: There is little or no detectable NCF1 and NOS2 expression in CD4 T cells in the spleen prior to clinical onset. a, a representative flow cytometry plot for CD4 T cells in the spleen. The splenocytes were collected from NCF1 deficient (Ncf1*/*.Nos2+/+) and sufficient mice (Ncf1+/+.Nos2+/+) at day 4 post immunization. The frequency and cell number of CD4 T cells in the spleen are shown, upon stimulation with PMA. b, mean florescence intensities (MFIs) of DHR and DCF staining of CD4 T cells are shown, after these cells were incubated ex vivo with PMA, PMA and ionomycin or DMSO as the control. c, the MFIs of NCF1 and NOS2 staining in CD4 T cells are shown. The number of mice is 7 per group. **p < 0.01 and ***p < 0.001 as determined by the Mann-Whitney U test. [file 12974_2020_1789_MOESM4_ESM.tiff]

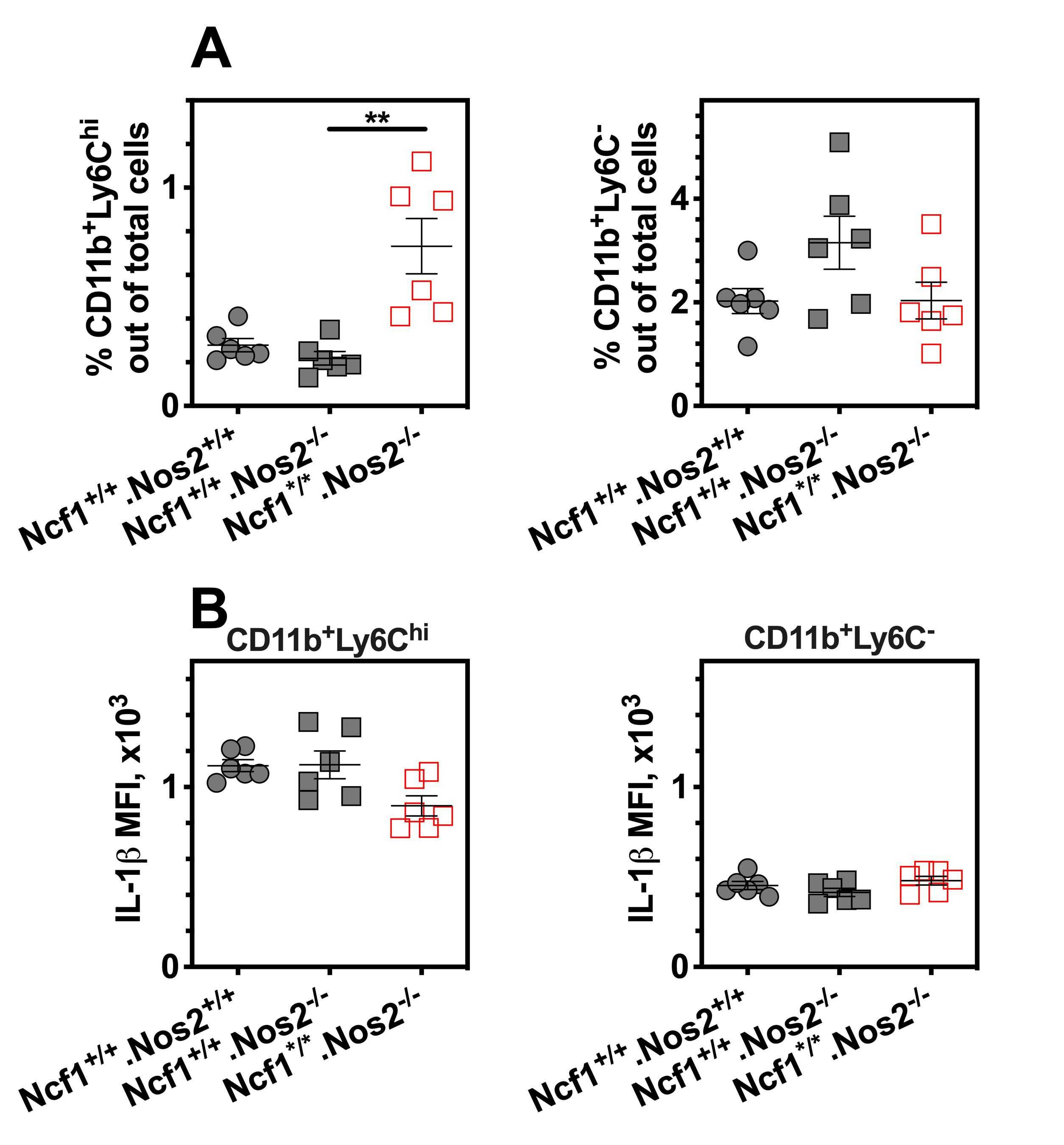

Supplement: Supplementary file 5 — Additional file 5: tiff format, title: There is no detectable change of pro-IL-1β expression in Ly6Chi monocytes and Ly6C- myeloid cells in the spleen prior to clinical onset. a, here are the frequencies of Ly6Chi monocytes and Ly6C- myeloid cells stated in Fig. 4a, upon stimulation with PMA. b, the MFIs of IL-1β in selected subsets are shown. The number of mice per group is 6. **p < 0.01 as determined by the Mann-Whitney U test. [file 12974_2020_1789_MOESM5_ESM.tiff]
